# Suitability of Different Thermometers for Measuring Body Core and Skin Temperatures in Suckling Piglets

**DOI:** 10.3390/ani11041004

**Published:** 2021-04-02

**Authors:** Simone M. Schmid, Wolfgang Büscher, Julia Steinhoff-Wagner

**Affiliations:** 1 Institute of Animal Science, University of Bonn, 53115 Bonn, Germany; s.schmid@uni-bonn.de; 2 Institute of Agricultural Engineering, University of Bonn, 53115 Bonn, Germany; buescher@uni-bonn.de

**Keywords:** core temperature, infrared thermography, infrared thermometry, suckling piglet, surface temperature, tympanic membrane temperature

## Abstract

**Simple Summary:**

After birth, piglets’ temperatures usually drop some degrees because of low ambient temperatures in the stable. Piglets have no ability to increase their body temperatures during their first days of life, which can cause health issues if piglets are not appropriately cared for. Monitoring temperatures can, therefore, contribute to reducing impaired wellbeing and unnecessary losses. The most common method for assessing core temperatures is measuring rectally with a digital thermometer. This, however, takes time and requires securing of the animal, which is stressful. Therefore, the aim of this study was to determine whether other thermometers or thermometric devices, such as an infrared camera, can deliver results similar to the digital thermometer. For the measurements in newborn piglets, infrared ear thermometers, infrared forehead thermometers, and infrared laser thermometers were used, as it was assumed that these would deliver results fast and cause little distress in piglets. The results were compared to rectally measured temperatures and it was found that the temperatures measured in-ear correspond to a great extent to rectal temperatures and show little variation between measurements, while the other used devices can only give a rough estimate of the actual core temperatures.

**Abstract:**

Monitoring the temperature of piglets after birth is critical to ensure their well-being. Rectal temperature measurement is time-consuming, requires fixation of the animal and is stressful for piglets. This study aims to evaluate the effectiveness of infrared thermometry and thermography as compared to rectal temperatures. We investigated digital thermometers for rectal measurements, infrared ear thermometers, infrared forehead thermometers, infrared laser thermometers and an infrared camera during field trials with piglets aged 1–13 days. Temperatures differed between the left and right ear and ear base (*p* < 0.01), but not between temples. Three forehead and laser devices yielded different temperatures (*p* < 0.01). Temperatures assessed with a laser thermometer decreased with distance from the target (*p* < 0.01). The highest correlation observed was between the rectal and tympanic temperatures (r = 0.89; *p* < 0.01). For temperatures assessed with the camera, inner thigh and abdomen correlated most closely to core temperature (0.60 ≤ r ≤ 0.62; *p* < 0.01). Results indicate that infrared ear thermometry commonly used in humans is also suited for assessing temperature in piglets. The inner thigh and abdomen seem promising locations for estimating core temperature with an infrared camera, but this approach needs to be adapted to reduce time exposure and stress for the piglets to be used under practical conditions.

## 1. Introduction

Body temperature is an important assessment for the early detection of stress and diseases in pigs, which often come along with fever in older pigs. In newborn piglets, a more concerning issue is postnatal hypothermia, a decrease in body temperature following birth [1,2]. Due to the lower temperatures in the farrowing unit adjusted to the needs of the sows, piglets are subject to heat loss from their body surface via convection and radiation [3]. In very lightweight piglets, an extended period of postnatal hypothermia can occur, decreasing their mobility and vitality and severely endangering their chance of survival [4,5]. Studies have shown a correlation between birth weight, vitality scores, and temperature during a piglet’s first days of life and that smaller piglets are more prone to heat loss due to their proportionally larger body surface area [3,6,7]. Already core temperatures of about 34–35 °C are critical for newborn piglets [2,8,9]. Additionally, routine interventions usually performed in the first days of life, such as castration and teeth resection, cause distress in piglets and can have significant effects on their body temperatures [10,11,12].

As a severe body temperature drop occurs immediately after birth, monitoring the body temperature of piglets postnatally is especially crucial for detecting conditions that threaten their well-being and survival [1,6,13,14]. Although piglets’ ability to regulate temperatures usually develops quickly during the first days of life, it is nonetheless necessary to continuously monitor temperatures during the suckling period to prevent unnecessary losses, and ensure appropriate development, as especially low-birth weight piglets can experience a delayed recovery from hypothermia [6,14]. Rectal temperature measurement has been considered the gold standard in human and veterinary medicine [15,16], but this procedure is time-consuming and, especially for newborn piglets, stressful and invasive [17]. It has been discussed previously that mere handling is stressful for piglets [18,19,20] and that a faster procedure can significantly reduce distress [21,22]. Infrared technologies are now used frequently in human medicine [23,24] and have already been applied for measuring temperatures in livestock, as discussed previously [25,26,27]. As several technologies have also been applied in older piglets [28,29], they might also be suitable for measuring the body core and surface temperatures of piglets in a faster and less disturbing manner [30]. Especially suitable for assessing surface temperatures are the “thermal windows”, which are body parts with high blood perfusion that lack insulation as they are not covered by a hair coat, e.g., the eyes or ear bases, while this is less the case at “non-thermal windows” with a thicker fat layer, such as the shoulder [26,31,32]. However, not only does the targeted body location influence the results of the temperature assessment, but also external influences such as the ambient temperature and soiling of the body play a role [25,26,32,33]. This study aims to compare the accuracy of multiple thermometry and thermography devices for assessing body core and surface temperatures as compared to rectal temperature.

## 2. Materials and Methods

This study was conducted in accordance with federal and institutional animal use guidelines (Az. 81-PM EHaasb 05.11.18.15.15), the data privacy agreement (University of Bonn, 38/2018), and ethical standards. This study was subdivided into two parts: a first part consisting of a reliability and replicability study, where different devices were tested under the same conditions, and a second part, where the previously tested devices were applied under field conditions.

### 2.1. Reliability and Replicability Study

Before starting the field trials, the reliability of the thermometry and thermography devices (Table 1) was tested by replicated measurements in 6-day-old piglets with pink skin ((Landrace × Large White) × Pietrain) (except for the infrared forehead thermometer: piglet age 9–13 days) in conventional pig production farms in Germany. Piglets were kept with the sow and litter in individual farrowing crates with partly slatted flooring and had access to a piglet nest with a heat lamp. Mean ambient temperatures were 19.5 °C ± 3.5 °C. For the temperature measurements, piglets were removed from the crates individually. One person secured the piglet and measured the temperature with all devices except the laser thermometer and infrared camera, which were applied by a second person. The duration until a measuring result was displayed was determined by the applied device and could not be adjusted by the operator. The end of the measurement was indicated with a beeping sound by all devices. If adjustable, in particular for the infrared camera and laser devices, emissivity was set at 0.98 units. This value was chosen according to the information provided by the camera’s manufacturer for (human) skin [34] and previous reports regarding thermography in livestock [27,32,33]. As the thermographic camera measures the infrared radiation from the surface of an object, in this case the piglets, it is necessary to include the emissivity for a correct calculation and translation into temperatures [25,33]. The infrared camera calibrated automatically once starting the software; image sharpness was adjusted by manually rotating and focusing the camera’s objective.

The temperature of each piglet was measured using 3 different rectal thermometers, with 3 consecutive measurements taken for each device (Table 1). Likewise, temperature was measured in each ear using 3 different infrared ear thermometers (with disposable hygiene caps), with 3 consecutive measurements taken for each device. Temperatures were also measured with 3 different infrared forehead thermometers, with 3 replications for each. These contactless forehead thermometers were held approximately 3 cm from the piglet’s head. To assess the reliability of the infrared laser thermometer, 3 consecutive measurements were performed with 3 devices. The effect of distance was assessed by taking measurements at a distance of 10, 30, 50 and 100 cm from the piglet’s head. Furthermore, 3 consecutive analyses of the thermographic images taken with the infrared camera were performed. However, it should be noted here that not all devices were applied in every piglet; hence, different numbers of measurements arise (Table 1). The number of measurements further depends on the number of measuring locations, e.g., 270 measurements performed with the infrared forehead thermometer result from measuring at 3 body locations (left, right and middle forehead), from 1 distance (5 cm), with 3 devices and 3 repetitions per device in 10 piglets: 3 × 1 × 3 × 3 × 10 = 270 measurements. For the infrared laser thermometer, temperatures were measured in 10 piglets from a distance of 10 cm, while in 5 of these piglets, additional measurements were taken from 30, 50 and 100 cm, resulting in a total measurement number of 450.

### 2.2. Testing Thermometers’ Suitability during Field Trials

In a separate trial, the methods described in Section 2.1 were tested under field conditions; for this, the same devices as applied and specified in the previous reliability study were used. Temperature measurements were performed in the farrowing units of 4 conventional farms. Temperatures were assessed in a total of 403 piglets. As in the reliability trial, piglets were kept in farrowing crates with the dam and littermates on partly slatted flooring, with access to a piglet nest warmed by an infrared lamp. The mean ambient temperature was 20.2 °C ± 1.6 °C. The piglets, aged 1–7 days with pink skin ((Landrace × Large White) × Pietrain), were picked up individually and secured during the measurements. Rectal temperatures were measured using a digital thermometer (*n* = 958). Tympanic membrane temperatures were measured using an infrared ear thermometer (*n* = 424), while skin temperatures were assessed using an infrared laser thermometer at the ear base (*n* = 671). Infrared images were taken with the camera at 6 locations on the body (head, throat, ribs, hip, inner thigh, abdomen) (*n* ≥ 488). The number of measurements taken differed between devices because time limitations precluded the use of all devices at all farms; however, for all assessed infrared temperatures, a rectal value measured at the same time is available for comparison. First, tympanic temperatures and temperatures at the ear base were assessed, followed by assessment with the infrared camera and rectal measurements.

For temperature assessment with the infrared camera, a test bench was set up in the stable. A black mat was placed on a table to provide a uniform background. The camera, attached to a tripod and a laptop computer, was also situated on the table. The camera was adjusted at an angle of approximately 15° perpendicular to the table and the laying piglet so that it could capture the whole body of the piglet. The piglets were secured on the mat by holding their legs. The person handling the piglets wore disposable rubber gloves for hygienic reasons and to mitigate heat transfer to the pig’s body. The distance between the camera and piglet was approximately 50 cm. Two 10-s videos of each piglet were recorded, one while lying on its side and one while lying on its back. Thermographic recordings were analyzed using Optris PIX Connect software (Rel. 3.2.3023.0) when the piglets were in an ideal position. On the image, squares of 15 × 15 mm width and height were positioned on the 6 chosen measuring areas; the minimum, median, and maximum temperatures were assessed.

### 2.3. Statistical Analysis

Data collected during trials were transferred to an Excel spreadsheet and coefficients of variation (CV) were calculated (Excel 2016, Microsoft, Redmond, WA, USA). Statistical analysis was performed with SAS system 9.4 (SAS Institute Inc., Cary, NC, USA) for both the reliability study and the field trials. Data were compared between individual measurements, individual devices, measuring locations, distances from measuring location and body sides. Comparisons between these factors were performed with the Kruskal–Wallis test and the Wilcoxon signed-rank test by applying the PROC NPAR1WAY procedure, for which either individual measurement, individual device, measuring location, distance from measuring location or body side were entered as a class. Furthermore, the Spearman rank correlation (PROC CORR spearman) procedure was used to determine correlations between rectal temperatures and the temperatures measured by the other devices investigated during the reliability study and the field trials. Linear regression analysis and receiver operating characteristic (ROC) curve analysis were performed with SigmaPlot 14.0 (Systat Software Inc., San José, CA, USA). ROC curves were generated to assess the performance of the different thermometers when using a cut-off point of 39.2 °C for rectally measured temperatures. For this, sensitivity, specificity and area under the curve (AUC) values were calculated with SigmaPlot. The level of significance was set at *p* < 0.05, while *p* < 0.01 was regarded as highly significant and *p* < 0.1 as a tendency. Data are presented as the mean ± SD.

## 3. Results

### 3.1. Testing Devices for Reliability and Replicability

The measuring of rectal temperatures was the most time-consuming procedure, requiring about 15 s per measurement. All infrared thermometers (ear, forehead, and laser) required less than 3 s per measurement. When investigating the reliability and replicability of the thermometers, we observed that the three consecutive temperatures measured with one rectal thermometer (CV < 0.01) as well as the temperatures measured with different devices (means: 38.95 °C ± 0.23 °C, 38.87 °C ± 0.30 °C and 38.91 °C ± 0.34 °C) did not differ. No differences and a low CV (0.001 < CV < 0.013) were detected for three consecutive body temperatures measured with the same infrared ear thermometer. Consecutive measurements in the left and the right ear yielded similar results. Temperatures were not different between the different ear thermometers (means: 38.76 ± 0.78 °C, 38.90 ± 0.71 °C, 38.87 ± 0.73 °C). However, the temperatures measured in the right ear (38.51 °C ± 0.70 °C) were significantly lower than those measured in the left ear (39.17 °C ± 0.63 °C) (*p* < 0.01). Repeated measurements with the infrared forehead thermometers did not differ (0.001 < CV < 0.038). Temperatures differed significantly between the three different infrared forehead devices (*p* < 0.01), with one device measuring higher temperatures (38.73 ± 0.90 °C) than the other two (38.26 ± 0.73 °C, 38.35 ± 0.97 °C). For temperatures measured at the right (38.36 ± 0.91 °C), left (38.57 ± 0.99 °C), and middle (38.40 ± 0.77 °C) of the forehead, no differences were detected. Additionally, for repeated measurements using thermographic images, no differences were observed.

**Figure 1:**
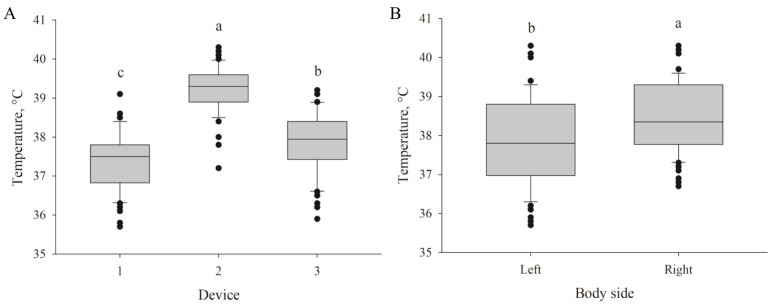
Skin temperatures (*p* < 0.001) measured with 3 laser thermometers at the same measuring point at both sides of the body of piglets (*n* = 180) at 10 cm distance (**A**) and differences of temperatures (*p* < 0.001) measured at left and right side of the body of newborn piglets (*n* = 180) at 10 cm distance with the same infrared laser thermometer (**B**). Different letters (a, b, c) indicate significant differences.

Significant differences were observed between measurements taken with the infrared laser thermometers. Although temperatures measured repeatedly with the same device from a 10-cm distance did not differ significantly on either body side (CV < 0.01), significant differences were observed between temperatures measured using different devices (37.40 °C ± 0.75 °C, 39.21 °C ± 0.59 °C, and 37.88 °C ± 0.83 °C) (*p* < 0.01) (Figure 1A).

**Figure 2:**
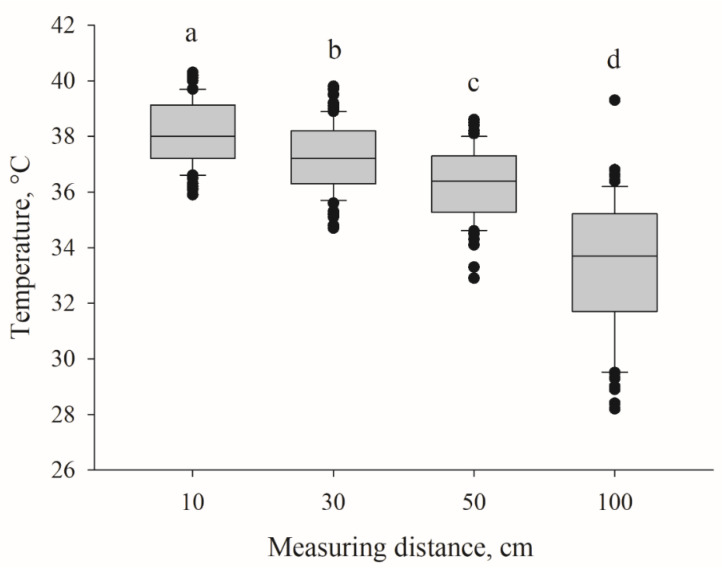
Span of skin temperatures measured with distances of 10, 30, 50 or 100 cm and differences (*p* < 0.01) between infrared laser thermometer and piglet (*n* = 360). Different letters (a, b, c, d) indicate significant differences.

The temperature measured from a 10-cm distance also differed significantly between the left and right ear base (mean, 37.86 ± 1.11 °C vs. 38.47 °C ± 0.91 °C, respectively) (*p* < 0.01) (Figure 1B). However, this difference was not detectable when including temperatures from all distances (10–100 cm). The temperature decreased significantly with increasing distance between the device and target (*p* < 0.01). The mean temperature at a distance of 10 cm was 38.17 °C ± 1.06 °C but only 33.29 °C ± 2.38 °C at 100 cm (Figure 2).

### 3.2. Comparing Temperature Assessment Methods during Field Trials

**Figure 3:**
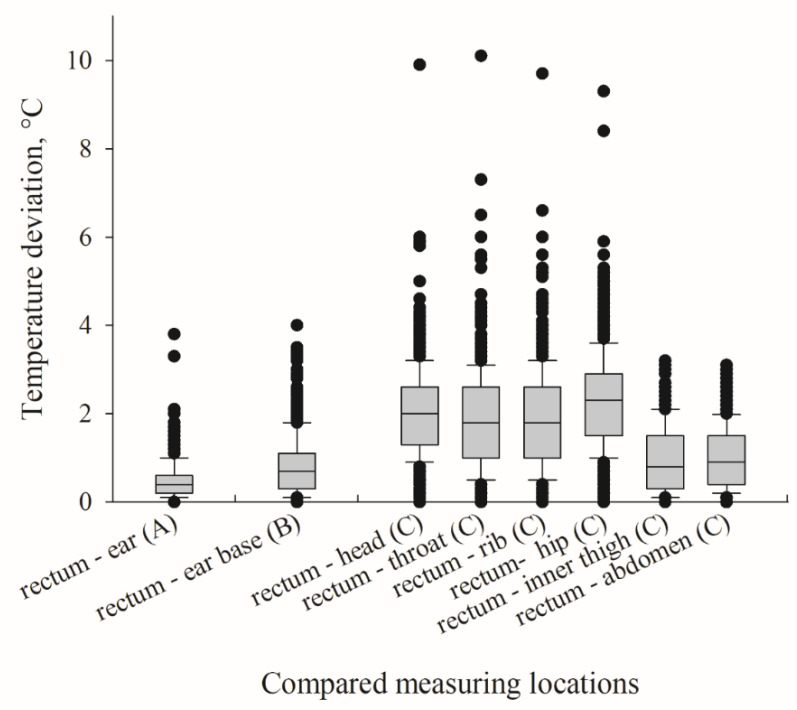
Temperature deviations as a difference between the gold standard (rectal temperatures) and temperatures assessed in-ear (**A**: infrared ear thermometer, *n* = 423), at ear base (**B**: infrared laser thermometer, *n* = 670), and at head, throat, rib, hip, inner thigh, and abdomen with an infrared camera in piglets (**C**: *n* ≥ 488). Differences were calculated per pig as absolute amounts (∆t = |t_rectal_ − t_x_|).

Temperature deviations and differences between rectal thermometer assessment and infrared ear thermometer, laser thermometer, and infrared camera are shown in Figure 3. The smallest difference and deviation from the rectal assessment was observed for in-ear temperature measurements, followed by those assessed at the ear base using the infrared laser device (95% quantile, 1.3 °C and 2.1 °C, respectively). Temperatures assessed with the infrared camera at the inner thigh and abdomen deviated little from rectal temperatures (95% quantile, 2.4 °C and 2.2 °C), while a greater difference from the rectal temperature assessment was observed for those assessed on the head, throat, ribs, and hip (95% quantile ≥ 3.5 °C).

**Figure 4:**
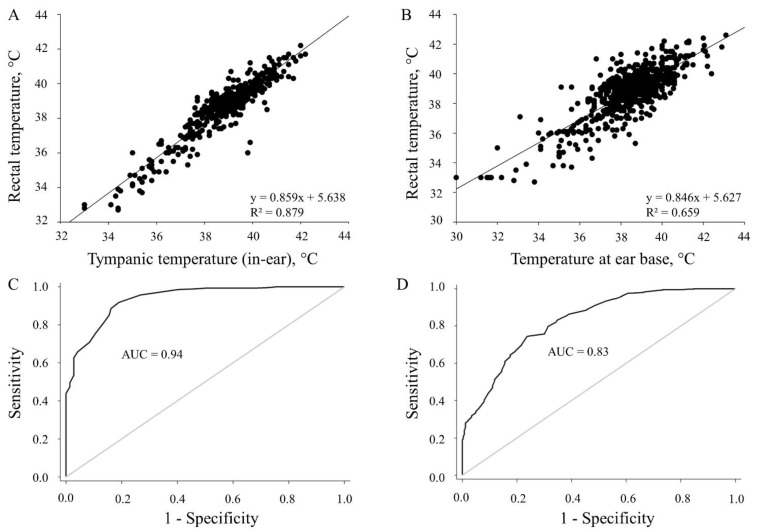
Scatter plots (**A**,**B**) and ROC curves including the area under the curve (AUC) (**C**,**D**) showing correlations and comparing rectally measured temperature with a digital thermometer and temperatures assessed using (**A**,**C**) an infrared ear thermometer (*n* = 424) or (**B**,**D**) an infrared laser thermometer (*n* = 671) in piglets during field trials (*p* < 0.01).

All temperatures assessed using thermometry and thermography methods correlated positively with rectal temperatures (*p* < 0.01). Correlations of rectally measured temperatures with temperatures assessed with infrared ear and infrared laser thermometers are shown in Figure 4, as are the regression equations and coefficients of determination. The AUC was 0.94 obtained for the infrared ear thermometer (95% CI: 0.91–0.96) and 0.83 for the infrared laser thermometer (95% CI: 0.79–0.86) at a cut-off point of 39.2 °C (Figure 4). The AUC for the assessment with the infrared camera ranged from 0.72 to 0.75. The respective correlation coefficients are shown in Table 2; only those for the maximum values are presented. The correlation was highest between the digital rectal thermometer and the infrared ear thermometer (r = 0.89; *p* < 0.01) and between the digital rectal thermometer and the infrared laser thermometer (r = 0.69; *p* < 0.01), and moderate between the digital rectal thermometer and the infrared camera (0.41 ≤ r ≤ 0.62; *p* < 0.01). Of the thermographically assessed temperatures, the highest correlation to rectal temperature was that assessed at the inner thigh (r = 0.62; *p* < 0.01).

## 4. Discussion

The experiments undertaken in the course of the present study show that not all of the applied techniques and devices are suitable for generating reliable and exact results that are comparable to rectal temperatures. Results have revealed high correlations between rectal and tympanic temperatures. Correlations between rectal temperatures and those measured at the forehead and ear base were lower; additionally, the individual infrared forehead and laser thermometers that were applied delivered varying results. The larger the distance was between the piglet and laser thermometer, the less exact the displayed temperature was and the higher the resulting temperate span was, indicating a lower quality of the measured values. When interpreting the results of the present study, it should be considered that not all devices were applied in all trial piglets and that piglets varied in age. Additionally, a valuable approach for future studies would be to assess the behavior (i.e., defense movements) of piglets during the measurements with the different thermometers to further describe the impact of using these devices on animal welfare.

Temperature measurement can induce stress in piglets. Nonetheless, temperature monitoring in piglets is important to avoid losses due to hypothermia or illness. The obtained temperatures can serve as warning signs to stock persons indicating a possible health issue [35]. While temperature measurement using a digital rectal thermometer is standard practice, this method is time-consuming. The digital thermometer used in this study is supposed to display a result within 9 s but required approximately 15 s per measurement. Additionally, use of the rectal thermometer requires that each animal is picked up and secured, which takes time as well and induces stress. In this study, several temperature measurements were performed consecutively, which might have enhanced the core temperatures to some extent. Furthermore, defense movements during this invasive procedure can cause injuries, and urination and defecation can impede the process.

Our study results indicate that the infrared ear thermometer is a suitable alternative for assessing temperature in piglets. The correlation coefficients between the rectal and infrared ear devices were high during the field trials. Additionally, tympanic measurements showed little variation compared to the other evaluated devices, which is important to consider. An advantage of the infrared ear thermometer is its ease of use. The in-ear measurements required much less time to assess than the rectal measurements did, which is a critical factor for reducing stress in piglets [21]. Another advantage of infrared thermometers is their quick measurement display.

Several concerns have been raised regarding the use of infrared thermometers in animals. First, the use of this method might be limited if acute otitis media affects tympanic temperatures. However, human and veterinary medicine studies have demonstrated that such infection does not affect tympanic temperature [36,37]. Second, Sousa et al. [15] reported limited agreement between the rectal and auricular temperatures in dogs and assumed that inadequate positioning of the thermometer made for human ears in the dog ears might have led to these results. The infrared ear thermometers used in the present study were also designed for use in humans, but seemed to fit the piglet ears without problems. Additionally, securing the piglet and taking the measurement were easily performed by one person. In human studies, it was concluded that accurate results can be obtained without intensive training [36]. Nonetheless, operator effects may have been present in the present study, as a difference of about 1 °C was observed between measurements in the left and right ear. The same was observed in human ears as summed up by Levander and Grodzinsky [38]. During the procedure, the piglets were held with the left hand while the temperature was measured with the right hand. To access the right ear, the operator had to reach over the piglet and might have inserted the thermometer at a different angle than in the left ear. The contact between the measurement sensor and tympanic membrane may have been lower in the right ear, resulting in lower temperatures. Additionally, the manufacturer’s information sheet delivered with the infrared ear thermometer includes a note that measurement differences between left and right ear are naturally occurring. Another explanation for the detected differences could also be anatomical variances on the two body sides, but, as no evidence for a physiological difference in temperature between the ears could be found, the operator effect is the more probable cause [39].

Although the in-ear measurement was less invasive than rectal measurement, the proximity of the operator’s hands and the measuring device to the head seemed to bother some piglets. Comparison of piglet responses to the different devices was not part of the present study, but we observed short stress-related movements and vocalizations in most piglets in response to being picked up and secured, regardless of the device used. Future studies assessing piglet behavior during measurements with different thermometers are recommended to confirm the findings of the present investigation.

Cross-contamination is a risk with the use of rectal and ear thermometers. Therefore, contact thermometers should be wiped and disinfected after measuring each piglet, and the disposable hygiene cups should be used with the ear thermometer to prevent contamination. This risk is absent in infrared laser thermometers, as they enable contactless measurement [26]. The difference between the two contactless thermometers used in the present study is that the forehead thermometer is specifically developed for measuring temperatures on the human forehead and uses this measurement to calculate the core temperature. In contrast, the laser thermometer, does not convert the measured value into core temperature. According to Sethi et al. [40], the human forehead is an optimal area for temperature measurements due to its high blood supply. This is transferable to piglets, who are born with a soft coat and, unlike older piglets, do not have bristles that can distort the measurements. Previously, high correlations between forehead and rectal temperatures were detected in cattle [41]. However, limitations of forehead thermometry are that the temperature of the forehead can be influenced by a varying perfusion and external effects [42] and that frequent head movements can distort the results [27]. Nonetheless, the handling and application of the forehead thermometer was easy and rapid in the present study. This device affords flexibility, as measurements did not differ between the left and right temples and the middle forehead. However, we observed that the correlation between the temperatures measured with this device and the rectal thermometer was only moderate, and several pediatric studies report that this thermometry method is less reliable than tympanic measurements [16,43]. Nonetheless, measuring forehead temperature could be less stressful to piglets, as this method is even less invasive than ear thermometers and should be further investigated in field trials. The observation that one of the three tested devices gave different results than the others suggests that other models should be considered for field trials and that devices should not be exchanged within experiments. Although the difference was less than 0.5 °C, this factor should be taken into consideration in future experiments. As explained by Burnham et al. (2006), different thermometer models might generate different results; therefore, the current findings should be interpreted with caution.

Each temperature assessment device has benefits and drawbacks. Securing piglets is necessary even if using contactless thermometers, as any motion can skew the measurements [27]. Additionally, the skin temperature is constantly influenced by its surroundings [33] and can, therefore, vary more than core temperatures as was also shown by our data. When using the infrared laser thermometer, external factors such as the ambient temperatures, the heat source in the piglet nest, humidity or increased air speed can influence the skin temperature [26,44,45]. As there are usually different temperature zones in farrowing crates, piglet skin temperatures can be influenced by their respective location in the crate. Additionally, pig skin color could play a role [30], which needs to be further investigated. Furthermore, the temperature assessed with a laser thermometer developed for industrial purposes does not represent the actual core temperature, so measurements must be interpreted with caution.

We observed that the greater the distance of the device from the piglet, the lower the measured temperature and the higher the variation in measured temperatures. From a greater distance, a larger area is assessed by the laser, which could also explain why we found a difference between skin temperatures at the left and right ear base when measured from a short distance, but not from a larger one. While assessing temperatures at a greater distance saves time, the decrease in accuracy with distance suggests that assessing the temperature of a piglet lying a few meters into the pen is not a suitable option. At greater distances, aiming accurately at the desired measuring point is difficult. Results of a pretest to the present study revealed that skin temperature measured with a laser thermometer at the ear base correlated more closely to rectally measured temperatures than skin temperatures of the forehead or flank did. Correlations between temperatures measured at the lower belly and rectum were also promising, but the ear base seemed more easily accessible than the lower belly. This observation is consistent with that of Soerensen and Pedersen [26], who reported that while the ear base is a thermal window that indicates the body temperature, the skin temperature at other locations of the body might be lower. Nonetheless, laser thermometers are advantageous with respect to the rapidity and non-invasiveness of the measurements. As with infrared forehead thermometers, the three laser devices assessed in this study generated different temperatures, confirming observations made by Ng et al. [46].

While previous studies report that thermographic images can serve as early indicators of health issues, limitations to this technique, such as dirt or moisture on the body surface, have been mentioned [25,45,47]; further research is needed before its reliable use is possible [26]. Naturally, piglets might come into contact with feces or water, which could affect skin temperature readings. As shown previously [45], maximum temperature values had the lowest variance and were less influenced by animal soiling hence they were also used here. As with the infrared laser thermometer, the distance between the infrared camera and the target influences the temperature values [48]. Therefore, a standardized test bench was set up in the current study to provide stability. Using the infrared camera, it was possible to assess minimum to maximum temperatures in several body parts in one image. Temperatures measured at the inner thigh correlated most closely to rectal temperatures, probably due to fewer external effects, as well as the thinner fat layer and thinner skin with finer hair in this body area.

The infrared camera used in this study required additional gear (a laptop and cables) for the measurements. The set-up and measurements are time-consuming and seemed to cause distress in the piglets, who were secured on the table for a few seconds during the recording. Further research is needed to develop standardized set-ups for recording thermal images without disturbing the piglets. While infrared thermometers and cameras have the potential to measure temperatures without contact and, therefore, to prevent stress in animals [30], our results suggest that the measurements are less reliable than other devices. In addition, the infrared camera technology is very expensive and therefore, not suited for daily use under practical conditions. An alternative might be the use of cell phones with integrated infrared cameras or camera attachments, as these are more practical. However, the suitability of these technologies should be verified before usage.

## 5. Conclusions

Several factors can influence the accuracy of temperature measurements, which need to be considered when generating and interpreting results. The findings of this study suggest that the infrared ear thermometer seems suited for assessing temperatures in piglets, as it is reliable and provides temperature values equal to those of rectal thermometers. However, fixation of the animal is still required. The infrared forehead thermometer is a flexible and non-invasive measuring technique but its suitability should be further evaluated in field trials. The infrared laser thermometer could be used to assess body temperature at a short distance, but its limited reliability should also be considered. To assess piglet body temperature with the infrared camera, the inner thigh and abdomen seem to be promising measurement locations. These results should be verified in future studies by taking the discussed influences into account, so that the devices’ suitability for improving health monitoring can be confirmed.

## Figures and Tables

**Table 1:** Overview of repeated measurements to evaluate the reliability of different thermometers for assessing temperatures in piglets.

Device	Temperature Assessed	Measurement Location	Measuring Distance	Number of Tested Devices	Number of Repetitions/Device	Number of Piglets	Total Number of Measurements
Digital rectal thermometer (Geratherm GT-195-1)	Core temperature	Rectum	Direct contact	3	3	10	90
Infrared ear thermometer (ThermoScan IRT6520)	Core temperature	Middle ear (left; right)	Direct contact	3	3	10	180
Infrared forehead thermometer (Jumper JPD-FR202)	Core temperature	Forehead (left; right; middle)	5 cm	3	3	10	270
Infrared laser thermometer (Eventek ET312)	Skin temperature	Ear base (left; right)	10 cm (10 piglets), 30, 50, 100 cm (5 piglets)	3	3	10	450
Infrared camera (Optris PI400)	Skin temperature	Head, throat, rib, hip, inner thigh, abdomen	50 cm	1	3	15	270

**Table 2:** Correlation coefficients (r) of thermometry and thermography methods to rectal temperatures (gold standard, bold letters) and to each other investigated in piglets (*n* ≥ 163) aged 1–7 days (*p* < 0.01).

Device/ Measuring Location	Infrared Ear Thermometer	Infrared Laser Thermometer: Ear Base, 10 cm	Infrared Camera: Head	Infrared Camera: Throat	Infrared Camera: Rib	Infrared Camera: Hip	Infrared Camera: Inner Thigh	Infrared Camera: Abdomen
Digital thermometer	0.89	0.69	0.53	0.48	0.46	0.51	0.62	0.60
Infrared ear thermometer		0.74	0.63	0.66	0.64	0.68	0.73	0.62
Infrared laser thermometer: ear base, 10 cm			0.64	0.68	0.64	0.64	0.62	0.65
Infrared camera: head				0.87	0.86	0.80	0.82	0.78
Infrared camera: chest					0.95	0.88	0.84	0.80
Infrared camera: rib						0.90	0.84	0.80
Infrared camera: hip							0.82	0.79
Infrared camera: inner thigh								0.86

## Data Availability

The data presented in this study are available on request from the corresponding author.

## References

[1] Tuchscherer M., Puppe B., Tuchscherer A., Tiemann U. (2000). Early identification of neonates at risk: Traits of newborn piglets with respect to survival. Theriogenology.

[2] Pedersen L.J., Malmkvist J., Kammersgaard T., Jørgensen E. (2013). Avoiding hypothermia in neonatal pigs: Effect of duration of floor heating at different room temperatures. J. Anim. Sci..

[3] Vande Pol K.D., Tolosa A.F., Shull C.M., Brown C.B., Alencar S.A.S., Ellis M. (2020). Effect of drying and/or warming piglets at birth on rectal temperature over the first 24 h after birth. Transl. Anim. Sci..

[4] Caldara F.R., Dos Santos L.S., Machado S.T., Moi M., de Alencar Nääs I., Foppa L., Garcia R.G., Dos Santos R.D.K.S. (2014). Piglets’ surface temperature change at different weights at birth. Asian-Australas J. Anim. Sci..

[5] Baxter E.M., Jarvis S., Sherwood L., Robson S.K., Ormandy E., Farish M., Smurthwaite K.M., Roehe R., Lawrence A.B., Edwards S.A. (2009). Indicators of piglet survival in an outdoor farrowing system. Livest. Sci..

[6] Kammersgaard T.S., Pedersen L.J., Jørgensen E. (2011). Hypothermia in neonatal piglets: Interactions and causes of individual differences. J. Anim. Sci..

[7] Santiago P.R., Martínez-Burnes J., Mayagoitia A.L., Ramírez-Necoechea R., Mota-Rojas D. (2019). Relationship of vitality and weight with the temperature of newborn piglets born to sows of different parity. Livest. Sci..

[8] Mount L.E. (1959). The metabolic rate of the new-born pig in relation to environmental temperature and to age. J. Physiol..

[9] Lay D.C., Matteri R.L., Carroll J.A., Fangman T.J., Safranski T.J. (2002). Preweaning survival in swine. J. Anim. Sci..

[10] Bonastre C., Mitjana O., Tejedor M.T., Calavia M., Yuste A.G., Úbeda J.L., Falceto M.V. (2016). Acute physiological responses to castration-related pain in piglets: The effect of two local anesthetics with or without meloxicam. Animal.

[11] Lonardi C., Scollo A., Normando S., Brscic M., Gottardo F. (2015). Can novel methods be useful for pain assessment of castrated piglets?. Animal.

[12] Fu L.-L., Zhou B., Li H.-Z., Liang T.-T., Chu Q.-P., Schinckel A.P., Li Y., Xu F.-L. (2019). Effects of tail docking and/or teeth clipping on behavior, lesions, and physiological indicators of sows and their piglets. Anim. Sci. J..

[13] Baxter E.M., Jarvis S., D’Eath R.B., Ross D.W., Robson S.K., Farish M., Nevison I.M., Lawrence A.B., Edwards S.A. (2008). Investigating the behavioural and physiological indicators of neonatal survival in pigs. Theriogenology.

[14] Herpin P., Damon M., Le Dividich J. (2002). Development of thermoregulation and neonatal survival in pigs. Livest. Prod. Sci..

[15] Sousa M.G., Carareto R., Pereira-Junior V.A., Aquino M.C.C. (2011). Comparison between auricular and standard rectal thermometers for the measurement of body temperature in dogs. Can. Vet. J..

[16] Teller J., Ragazzi M., Simonetti G.D., Lava S.A.G. (2014). Accuracy of tympanic and forehead thermometers in private paediatric practice. Acta Paediatr..

[17] Ramis G., Sánchez P., Úbeda J.L. Validation study of thermographic camera: Preliminary results [poster]. Proceedings of the 9th European Symposium of Porcine Health Management (ESPHM).

[18] Leslie E., Hernández-Jover M., Newman R., Holyoake P. (2010). Assessment of acute pain experienced by piglets from ear tagging, ear notching and intraperitoneal injectable transponders. Appl. Anim. Behav. Sci..

[19] Noonan G.J., Rand J.S., Priest J., Ainscow J., Blackshaw J.K. (1994). Behavioural observations of piglets undergoing tail docking, teeth clipping and ear notching. Appl. Anim. Behav. Sci..

[20] Sinclair A.R.L., Tallet C., Renouard A., Brunton P.J., D’Eath R.B., Sandercock D.A., Prunier A. Behaviour of isolated piglets before and after tooth clipping, grinding or shamgrinding [abstract]. Proceedings of the 53rd Congress of the International Society for Applied Ethology (ISAE).

[21] Marchant-Forde J.N., Lay D.C., McMunn K.A., Cheng H.W., Pajor E.A., Marchant-Forde R.M. (2009). Postnatal piglet husbandry practices and well-being: The effects of alternative techniques delivered separately. J. Anim. Sci..

[22] Marchant-Forde J.N., Lay D.C., McMunn K.A., Cheng H.W., Pajor E.A., Marchant-Forde R.M. (2014). Postnatal piglet husbandry practices and well-being: The effects of alternative techniques delivered in combination. J. Anim. Sci..

[23] Craig J.V., Lancaster G.A., Taylor S., Williamson P.R., Smyth R.L. (2002). Infrared ear thermometry compared with rectal thermometry in children: A systematic review. Lancet.

[24] Burnham R.S., McKinley R.S., Vincent D.D. (2006). Three types of skin-surface thermometers: A comparison of reliability, validity, and responsiveness. Am. J. Phys. Med. Rehabil..

[25] Knizkova I., Kunic P., Gürdil G., Pinar Y., Selvi K. (2007). Applications of infrared thermography in animal production. Anadolu J. Agric. Sci..

[26] Soerensen D.D., Pedersen L.J. (2015). Infrared skin temperature measurements for monitoring health in pigs: A review. Acta Vet. Scand..

[27] Soerensen D.D., Clausen S., Mercer J.B., Pedersen L.J. (2014). Determining the emissivity of pig skin for accurate infrared thermography. Comput. Electron. Agric..

[28] Andersen H.M.-L., Jørgensen E., Dybkjær L., Jørgensen B. (2008). The ear skin temperature as an indicator of the thermal comfort of pigs. Appl. Anim. Behav. Sci..

[29] Loughmiller J.A., Spire M.F., Dritz S.S., Fenwick B.W., Hosni M.H., Hogge S.B. (2001). Relationship between mean body surface temperature measured by use of infrared thermography and ambient temperature in clinically normal pigs and pigs inoculated with *Actinobacillus pleuropneumoniae*. Am. J. Vet. Res..

[30] Schmidt M., Lahrmann K.-H., Ammon C., Berg W., Schön P., Hoffmann G. (2013). Assessment of body temperature in sows by two infrared thermography methods at various body surface locations. J. Swine Health Prod..

[31] Llamas Moya S., Boyle L.A., Lynch P.B., Arkins S. (2008). Surgical castration of pigs affects the behavioural response to a low-dose lipopolysaccharide (LPS) challenge after weaning. Appl. Anim. Behav. Sci..

[32] Sellier N., Guettier E., Staub C. (2014). A Review of Methods to Measure Animal Body Temperature in Precision Farming. Am. J. Agric. Sci. Technol..

[33] Kammersgaard T.S., Malmkvist J., Pedersen L.J. (2013). Infrared thermography--a non-invasive tool to evaluate thermal status of neonatal pigs based on surface temperature. Animal.

[34] Optris Infrared Sensing L.L. Basic Principles of Non-Contact Temperature Measurement. https://www.optris.com/downloads-infrared-cameras.

[35] Bressers H., te Brake J., Jansen M., Nijenhuis P., Noordhuizen J. (1994). Monitoring individual sows: Radiotelemetrically recorded ear base temperature changes around farrowing. Livest. Prod. Sci..

[36] Chamberlain J.M., Grandner J., Rubinoff J.L., Klein B.L., Waisman Y., Huey M. (1991). Comparison of a tympanic thermometer to rectal and oral thermometers in a pediatric emergency department. Clin. Pediatrics.

[37] González A.M., Mann F.A., Preziosi D.E., Meadows R.L., Wagner-Mann C.C. (2002). Measurement of body temperature by use of auricular thermometers versus rectal thermometers in dogs with otitis externa. J. Am. Vet. Med Assoc..

[38] Levander M.S., Grodzinsky E. (2017). Variation in Normal Ear Temperature. Am. J. Med. Sci..

[39] Childs C., Harrison R., Hodkinson C. (1999). Tympanic membrane temperature as a measure of core temperature. Arch. Dis. Child..

[40] Sethi A., Patel D., Nimbalkar A., Phatak A., Nimbalkar S. (2013). Comparison of forehead infrared thermometry with axillary digital thermometry in neonates. Indian Pediatrics.

[41] Salles M.S.V., Da Silva S.C., Salles F.A., Roma L.C., El Faro L., Bustos Mac Lean P.A., Lins de Oliveira C.E., Martello L.S. (2016). Mapping the body surface temperature of cattle by infrared thermography. J. Therm. Biol..

[42] Kistemaker J.A., den Hartog E.A., Daanen H.A.M. (2006). Reliability of an infrared forehead skin thermometer for core temperature measurements. J. Med. Eng. Technol..

[43] Hamilton P.A., Marcos L.S., Secic M. (2013). Performance of infrared ear and forehead thermometers: A comparative study in 205 febrile and afebrile children. J. Clin. Nurs..

[44] Hahn G.L., Eigenberg R.A., Nienaber J.A., Littledike E.T. (1990). Measuring physiological responses of animals to environmental stressors using a microcomputer-based portable datalogger. J. Anim. Sci..

[45] Lengling A., Alfert A., Reckels B., Steinhoff-Wagner J., Büscher W. (2020). Feasibility Study on the Use of Infrared Thermography to Classify Fattening Pigs into Feeding Groups According Their Body Composition. Sensors.

[46] Ng D.K., Chan C., Chan E.Y., Kwok K., Chow P., Lau W.-F., Ho J.C.-S. (2005). A brief report on the normal range of forehead temperature as determined by noncontact, handheld, infrared thermometer. Am. J. Infect. Control.

[47] Schaefer A.L., Cook N., Tessaro S.V., Deregt D., Desroches G., Dubeski P.L., Tong A.K., Godson D.L. (2004). Early detection and prediction of infection using infrared thermography. Can. J. Anim. Sci..

[48] Johnson S.R., Rao S., Hussey S.B., Morley P.S., Traub-Dargatz J.L. (2011). Thermographic Eye Temperature as an Index to Body Temperature in Ponies. J. Equine Vet. Sci..

